# Biological and other health related correlates of long-term life dissatisfaction burden

**DOI:** 10.1186/1471-244X-13-202

**Published:** 2013-08-01

**Authors:** Teemu Rissanen, Soili M Lehto, Jukka Hintikka, Kirsi Honkalampi, Tarja Saharinen, Heimo Viinamäki, Heli Koivumaa-Honkanen

**Affiliations:** 1 Department of Psychiatry, Kuopio University Hospital, P.O.B. 1777,FI-70211, Kuopio, Finland; 2 Department of Psychiatry, Päijät-Häme Central Hospital, Lahti, Finland; 3 School of Clinical Medicine, Psychiatry, University of Eastern Finland, Kuopio, Finland; 4 Institute of Clinical Medicine, Psychiatry, University of Tampere, Tampere, Finland; 5 School of Educational Sciences and Psychology, University of Eastern Finland, Joensuu, Finland; 6 Department of Psychiatry, South-Savonia Hospital District, Mikkeli, Finland; 7 Department of Psychiatry, North Karelia Central Hospital, Joensuu, Finland; 8 Department of Psychiatry, SOSTERI, Savonlinna, Finland; 9 Department of Psychiatry, SOTE, Iisalmi, Finland; 10 Department of Psychiatry, Lapland Hospital District, Rovaniemi, Finland; 11 Department of Psychiatry, Institute of Clinical Medicine, University of Oulu, Oulu, Finland

**Keywords:** Life satisfaction, Burden, Social support, Inflammation, Adiponectin, Metabolic syndrome

## Abstract

**Background:**

Mental health is interconnected with somatic health and can manifest itself in biological processes. Life dissatisfaction is an indicator of subjective well-being, but information on its biological correlates is scarce. The aim of this study was to investigate the biological correlates along with other health-related factors of long-term life dissatisfaction in a population-based sample.

**Methods:**

As part of the Kuopio Depression Study, health questionnaires were sent to a randomly selected population-based sample in 1998, 1999, and 2001. In 2005, among a clinically studied sub-sample (n = 305), the 7-year long-term life dissatisfaction burden was assessed by summing life satisfaction scores from previous health questionnaires. Several sociodemographic, health, health behavior, and biological factors were investigated in respect to their associations to categorized (low and high) and continuous (linear regression) life satisfaction burden score (higher values indicating dissatisfaction).

**Results:**

In the final linear regression model long-term life dissatisfaction burden was significantly associated with poor social support (B = 0.138; p < 0.001), marital status (i.e. living alone) (B = 0.049; p = 0.019), current smoking (B = 0.087; p < 0.001), poor sleep (B = 0.052; p = 0.001), use of statins (B = −0.052; p = 0.002) and lower serum adiponectin level (B = −0.001; p = 0.039) whereas association of metabolic syndrome was marginally nonsignificant (B = 0.029; p = 0.055).

**Conclusion:**

Long-term life dissatisfaction is associated with adverse health, health behavioral, and social factors, as well as with a decreased anti-inflammatory buffer capacity, all indicating close relationships between subjective well-being and somatic morbidity.

## Background

Subjective well-being [1] is one of the main dimensions of good mental health. In longitudinal studies, self-reported life dissatisfaction has shown to be an indicator of long-term health hazards, i.e. increased mortality [2], including suicides [3] and unintentional injuries [4], work disability [5], and diseases such as coronary heart disease [6]. Cross-sectionally, life satisfaction is positively related to health, self-perceived wealth [7,8], better psychosocial functioning [9], and especially with social support [8,10-13].

The association between mental health and biological processes, especially inflammation, has recently become an important research topic. Negative relationships with others and a lack of purpose in life have previously been linked with a higher level of inflammation, for instance with the serum level of proinflammatory cytokine IL-6 in population-based studies [14,15]. Poor sleep quality, impaired sleep [16,17], and chronic psychological stress have also been linked with inflammation [18-20]. On the other hand, stressful life changes, health stress, a high body mass index and poor quality of sleep have been related to lower life satisfaction among women [21]. However, better knowledge is needed of the link between inflammatory factors and life satisfaction [22].

Adiponectin and resistin are adipocytokines secreted by adipose tissue [23,24]. Adiponectin acts in an anti-inflammatory manner [25,26], in contrast to proinflammatory resistin, and more conventional proinflammatory cytokines such as interleukin (IL)-6 and tumor necrosis factor (TNF)-α. Inflammation and cytokine responses have previously been associated with psychological stress [19,20]. Lowered serum adiponectin levels have been observed in major depressive disorder [27], panic disorder [28], and schizophrenia [29]. Moreover, among elderly patients with major depressive disorder, serum levels of adiponectin were lower than in non-depressed subjects [30]. However, the possible biological alterations do not follow categories of psychiatric diagnosis. For example, cytokine alterations in different diagnostic categories have closely resembled each other. Thus, evaluation of the inflammatory state with regard to global well-being is needed. In an English longitudinal study on ageing, well-being was noted to relate to low central adiposity in men, whereas in women it was related to a low level of the inflammatory marker hs-CRP [22]. Another previous study has reported an association between childhood maltreatment and lowered serum adiponectin levels [31]. Health-related factors such as obesity, metabolic syndrome, coronary heart disease, and the risk of cancer [24,32-34] have also been linked with levels of adiponectin.

The aim of this study was to investigate the biological and other health-related correlates of the long-term life dissatisfaction burden in a population-based sample. This may help to improve understanding of the relationships between subjective well-being and somatic morbidity. Such understanding may offer a potential for early interventions and the prevention of adverse biological and physiological changes.

## Methods

This study was conducted as part of the four-phase Kuopio Depression Study (KUDEP) focusing on the mental health of general population adults aged 25 to 64 years in the province of Kuopio in Eastern Finland. The study protocol was approved by the Research Ethics Committee of Kuopio University Hospital and the University of Kuopio. All subjects provided written informed consent before entering the study.

In 1998, a random sample of 3004 participants was selected from the National Population Register and followed up in 1999 and 2001. The baseline sample in 1998 included 2050 participants (response rate 68%), the first follow-up sample (in 1999) 1722 participants, and the second follow-up sample (in 2001) included 1593 respondents. The follow-up questionnaire was only sent to those participants who responded at baseline or in 1999.

In the fourth study phase in 2005, a sub-sample (n = 427) of the participants was invited for clinical evaluation and laboratory testing [35]. The inclusion criteria for this sub-sample were based on self-reported adverse mental symptoms or features, i.e. depression measured with the 21-item Beck Depression Inventory (BDI) [36], alexithymic features measured with the 20-item Toronto Alexithymic Scale (TAS) [37-39], and life dissatisfaction measured with the 4-item life satisfaction scale (LS) [2,40]. The following cut-offs were used: BDI > 9 [36], TAS > 57 [39], and LS > 11 [2]. Half of the selected subjects in the present study belonged to the symptomatic group (n = 209; 48.9%) and half to the asymptomatic group (n = 218; 51.1%). There were no differences between the groups in the gender distribution (females: 58% vs. 56%; p = ns) or age (mean (SD): 55.3 (9.0) years vs. 56.3 (10.1) years; p = ns). The participation rate in interviews was 78% (n = 333). Twenty-eight cases provided inadequate data and were excluded. The final sample included 305 cases, of which 175 (57.4%) were females and 130 (42.6%) males.

In the questionnaire in 2005, participants reported their age, gender, marital status (married or living with a partner vs. unmarried, separated or divorced, widowed, other), alcohol use (≥2 times per week vs. less), and current smoking (yes/no). Subjectively assessed social support (good/normal vs. poor), quality of sleep (good/normal vs. poor), and the presence of previous physician-given diagnoses of coronary heart disease (yes/no), hypertension (yes/no), cancer (yes/no), and rheumatoid arthritis (yes/no) were reported. Metabolic syndrome is a cluster of risk factors that include abdominal obesity, hyperglycemia, hypertension, low HDL, and high triglycerides. It was diagnosed according to modified criteria of the National Cholesterol Education Program Adult Treatment Panel III (NCEP ATP III) as described elsewhere [41]. Additionally, information on the usage of oral corticosteroids (yes vs. no), statins (yes vs. no), and nonsteroidal anti-inflammatory drugs [NSAID (yes vs. no)] was obtained from the questionnaire, as well as the register of the nationwide Social Insurance Institute.

Life satisfaction was assessed with a self-reported 4-item life satisfaction scale (LS-4, range 4–20, higher scores indicating lower life satisfaction). It has been used in general and patient populations [2,3,8,42,43]. The study subjects rated their general interest and happiness in life, ease of living, and experiences of loneliness, respectively, with the following scores: 1 = very interesting/happy/easy/not at all lonely; 2 = fairly interesting/happy/easy; 3 = cannot say/missing data; 4 = fairly boring/unhappy/hard/lonely; 5 = very boring/unhappy/hard/lonely. Psychometric properties of the 4-item life satisfaction scale have been reported in more detail elsewhere [2,5,8,44].

A long-term life dissatisfaction burden score was calculated based on the sum of LS scores in the four follow-up assessments (1998, 1999, 2001 and 2005). The study subjects were split into two groups according to the median score: the long-term satisfied (range 16–33; n = 159; 52.1%) and the long-term dissatisfied (range 34–80; n = 146; 47.9%) for the purpose of examining crude group differences. The mean LS scores (95% confidence intervals) in the long-term satisfied group during the follow-up were as follows: 1998: 6.96 (6.77–7.16); 1999: 6.85 (6.61–7.09); 2001: 6.69 (6.48–6.91); 2005: 6.45 (6.24–6.67). The respective figures for the long-term dissatisfied group were: 1998: 12.68 (12.16–13.21); 1999: 12.43 (11.87–12.99); 2001: 12.16 (11.64–12.68) and 2005: 11.14 (10.58–11.71. To verify the results, groups were formed consisting of the “stable satisfied” (LS score 1998 ≤ 11 and LS score 2005 ≤ 11; n = 197) and the “stable dissatisfied” (LS score 1998 ≥ 12 and LS score 2005 ≥ 12; n = 51). In the multivariate linear regression analyses, continuous score for long-term life dissatisfaction burden was used (c.f. statistical methods).

The laboratory measurements took place in the accredited Kuopio University Hospital medical laboratory. The body mass index (kg/m^2^) was calculated from height and body weight measured in light clothing without shoes. Venous blood samples were drawn after a 12-h overnight fast. The samples were frozen after the blood draw, and stored at −80°C until run. The levels of IL-6 (pg/ml) and TNF-α (pg/ml) were analyzed by multiplexing with Bio-Plex Human Cytokine Panel 1, utilizing a Bio-Plex instrument based on Luminex xMAP technology (Bio-Rad Laboratories Inc., Hercules, California, USA). High-sensitivity C-reactive protein (hsCRP) was determined by an immunoturbidimetric method (Konelab CRP, code 981699; Thermo Electron Co., Finland). The samples were analyzed using a Konelab 60i clinical chemistry analyzer (Thermo Electron). Adiponectin and resistin were quantified with a human serum adipokine (Panel A) LincoPlex kit (Millipore, MA, USA) using a Bio-Plex Suspension Array System (Bio-Rad Laboratories Pty Ltd, Hercules, CA, USA). The assay conditions were controlled, standardized, and pre-optimized to ensure optimal repeatability and reproducibility of the assays. Furthermore, the kit instructions and instrument manuals were carefully followed. The assayed kits were from the same lot, which allows better control of inter-assay variability. The samples were measured as a batch at the end of the study. The analysis process has been reported more precisely in previous studies [31,45].

### Statistical methods

Data analysis was carried out using SPSS (version 17.0). The differences in proportions were examined with the Pearson chi-squared test for categorical variables. As continuous variables did not follow a normal distribution, the group differences were assessed with the Mann–Whitney U-test. Both Pearson and Spearman correlations were used to study associations between continuous variables.

A logarithmic transformation was done for continuous life dissatisfaction burden score (logLSburd) due to its non-normal distribution. The correlates of long-term life dissatisfaction burden were assessed with linear regression models. Firstly, the crude associations between long-term dissatisfied and sociodemographic and health-related factors, including inflammatory biomarkers were studied. Secondly, based on these bivariate analyses, the significant correlates were gathered into two age- and gender-adjusted multivariate linear regression models (model 1 and model 2) (method: enter) (Tables 1 and 2). Model 1 (Table 1) investigated the role of sociodemographic factors and health behavior (i.e. age, gender, marital status, social support, sleep, and current smoking). Model 2 (Table 2) investigated the role of various biological and health-related factors (i.e. age, gender, metabolic syndrome, use of statins, and serum adiponectin level). The final model (Table 3) included all the significant correlates of long-term life dissatisfaction from models 1 and 2 (i.e. marital status, sleep, social support, current smoking, serum adiponectin level, metabolic syndrome, and the use of statins). Since active cancer may affect the inflammatory balance (32), additional analyses were performed to assess the association between the cancer diagnosis and inflammatory markers.

**Table 1:** **Socio-demographic factors and health behavior as correlates of long-term life dissatisfaction burden**^**1) **^**in linear regression models**

**Variables**	**Bivariate model**^**2) **^**B (95% CI)**^**1)**^	**p-value**^**2)**^	**Multivariate model 1**^**3) **^**B (95% CI)**^**2)**^	**p-value**^**3)**^
Age	**−0.002 (−0.004–0.000)**	**0.014**	−0.001 (−0.002 – 0.001 )	ns
Gender (male)	0.018 (−0.016–0.051)	ns	0.022 (−0.007 – 0.051)	ns
**Marital status **_**(living alone)**_	**0.077 (0.031–0.124)**	**0.001**	**0.055 (0.014–0.096)**	**0.009**
**Social support (poor)**	**0.179 (0.133–0.225)**	**<0.001**	**0.144 (0.099–0.188)**	**<0.001**
**Sleep (poor)**	**0.081 (0.049–0.114)**	**<0.001**	**0.051 (0.021–0.080)**	**0.001**
**Smoking (yes)**	**0.111 (0.070–0.152)**	**<0.001**	**0.098 (0.060–0.136)**	**<0.001**
Alcohol consumption ≥2/week	0.022 (−0.021–0.065)	ns	Nim	Nim

^1)^Long-term life dissatisfaction burden score (logLSburden): sum of life satisfaction scores in 1998, 1999, 2001 and 2005 with logarithmic transformation.

^2)^Bivariate linear regression analysis separately for each variable.

^3)^Multiple adjusted linear regression model 1 (method: enter) included the following factors: age (continuous), gender (0 = female, 1 = male), marital status (0 = married/living in a partnership, 1 = living alone), social support (0 = good/normal, 1 = poor), sleep (0 = good, 1 = poor), and smoking (0 = no, 1 = yes).

Nim = not included in the model due to nonsignificant association in the bivariate model.

**Table 2:** **Biological factors and medication as correlates of long-term life dissatisfaction burden**^**1) **^**in linear regression models**

**Variables**	**Bivariate model**^**2) **^**B (95% CI)**	**p-value**^**2)**^	**Multivariate model 2**^**3) **^**B (95% CI)**	**p-value**^**3)**^
Age	**−0.002 (−0.004–0.000)**	**0.014**	−0.001 (−0.003 – 0.001)	ns
Gender (male)	0.018 (−0.016–0.051)	ns	0.029 (−0.005 – 0.064)	ns
**Metabolic syndrome (yes)**	**0.038 (0.004–0.073)**	**0.030**	**0.046 (0.010 – 0.081)**	**0.012**
**Use of statins (yes)**	**−0.062 (−0.100– -0.023)**	**0.002**	**−0.064 (−0.106 – -0.023)**	**0.002**
**Adiponectin, [μg/ml]**	**−0.001 (−0.002–0.000)**	**0.016**	**−0.001 (−0.002 – 0.000)**	**0.023**
Coronary heart disease (yes)	−0.004 (−0.064 – 0.057)	ns	Nim	Nim
Rheumatoid arthritis (yes)	0.041 (−0.025 – 0.106)	ns	Nim	Nim
Hypertension (yes)	0.016 (−0.020 – 0.052)	ns	Nim	Nim
Use of oral corticosteroids (yes)	0.056 (−0.042 – 0.153)	ns	Nim	Nim
Use of NSAIDs (yes)	−0.003 (−0.096 – 0.090)	ns	Nim	Nim
BMI, [kg/m^2^]	0.003 (0.000–0.006)	ns	Nim	Nim
Resistin, [ng/ml]	0.000 (−0.002 – 0.002)	ns	Nim	Nim
hs-CRP, [mg/l]	−0.001 (−0.004 – 0.002)	ns	Nim	Nim
TNF-α, [pg/ml]	0.000 (0.000–0.000)	ns	Nim	Nim
IL6, [pg/ml]	0.000 (0.000–0.001)	ns	Nim	Nim

^1)^Long-term life dissatisfaction burden score: sum of life satisfaction scores in 1998, 1999, 2001 and 2005 with logarithmic transformation.

^2)^Bivariate linear regression analysis separately for each variable.

^3)^Multiple adjusted linear model 2 (method: enter) included the following factors: age (continuous), gender (0 = female, 1 = male), metabolic syndrome (0 = no, 1 = yes), use of statins (0 = no, 1 = yes), and serum adiponectin level (continuous).

Nim = not included in the model due to nonsignificant association in the bivariate model.

*Abbreviations*: *NSAID* Non-steroidal anti-inflammatory drug, *BMI* Body mass index, *hs-CRP* High-sensitivity C-reactive protein, *TNF-α* Tumor Necrosis Factor α, *IL6* Interleukin 6.

**Table 3:** **The association between long-term life dissatisfaction burden**^**1) **^**and its significant correlates in the final multivariate linear regression model**

**Variables**	**Final model**^**2) **^**B (95% CI)**	**p-value**
**Marital status**	**0.049 (0.008 – 0.090)**	**0.019**
**Sleep (poor)**	**0.052 (0.022 – 0.081)**	**0.001**
**Social support (poor)**	**0.138 (0.094 – 0.183)**	**<0.001**
**Smoking (yes)**	**0.087 (0.049 – 0.124)**	**<0.001**
**Adiponectin, [μg/ml]**	**−0.001 (−0.002 – 0.000)**	**0.039**
Metabolic syndrome (yes)	0.029 (−0.001 – 0.059)	ns (0.055)
**Use of statins (yes)**	**−0.052 (−0-086– -0.019)**	**0.002**

^1)^Long-term life dissatisfaction burden score: sum of life satisfaction scores in 1998, 1999, 2001 and 2005 with logarithmic transformation.

^2)^The final linear regression model (method: enter) included all the significant factors from models 1 and 2: marital status (0 = married/living in a partnership, 1 = living alone), sleep (0 = good, 1 = poor), social support (0 = good, 1 = poor), smoking (0 = no, 1 = yes), serum adiponectin level (continuous), metabolic syndrome (0 = no, 1 = yes), and use of statins (0 = no, 1 = yes).

## Results

The respondents in the long-term dissatisfied group reported more often living alone, experiencing poor social support and poor sleep, and were more often current smokers compared to the long-term satisfied (Table 4). Metabolic syndrome was more prevalent, but the use of statins less frequent among the long-term dissatisfied. No significant differences were found in the use of NSAIDs or oral corticosteroids, in gender, alcohol consumption, or the prevalence of coronary heart disease, rheumatoid arthritis, or cancer with respect to the long-term life dissatisfaction burden.

**Table 4:** Sociodemographic, health behavior, and biological factors in 2005 by two-category long-term life dissatisfaction burden score

	**Long-term life dissatisfaction burden**^**1)**^				
	**Satisfied (16–33 p.) N = 159**		**Dissatisfied (34–80 p.) N = 146**		
**Variables**	**Col%**	**(n)**	**Col%**	**(n)**	**p-value**
Gender (male), n (%)	42.1	(67)	43.2	(63)	ns
Living alone, n (%)	7.5	(12)	21.9	(32)	**<0.001**
Smoking (yes), n (%)	8.2	(13)	29.5	(43)	**<0.001**
Alcohol consumption ≥2/week, n (%)	16.4	(26)	19.9	(29)	ns
Social support (poor)	1.3	(2)	24.1	(36)	**<0.001**^**3)**^
Sleep (poor), n (%)	42.8	(68)	69.2	(101)	**<0.001**
Metabolic syndrome, n (%)^2)^	31.2	(48)	43.4	(63)	**0.028**
Hypertension, n (%)	28.9	(46)	32.2	(47)	**ns**
Coronary heart disease, n (%)	7.5	(12)	8.9	(13)	ns
Rheumatoid arthritis, n (%)	6.3	(10)	7.5	(11)	ns
Cancer, n (%)	1.3	(2)	2.7	(4)	ns^3)^
Use of oral corticosteroids, n (%)	2.5	(4)	3.4	(5)	ns^3)^
Use of NSAIDs, n (%)	4.4	(7)	2.1	(3)	ns^3)^
Use of statins, n (%)	30.2	(48)	16.4	(24)	**0.005**
**Variables**	**Mean (95% CI)**		**Mean (95% CI)**		**p-value**
**Age**^**4)**^	56.9 (55.3–58.6)		55.1 (53.6–56.6)		**0.025**^5)^
BMI, [kg/m^2^]^4)^	27.1 (26.4–27.9)		27.8 (26.8–28.8)		ns^5)^
**Adiponectin**, [μg/ml]^4)^	18.39 (15.59–21.19)		13.38 (11.44–15.31)		**0.014**^5)^
Resistin, [ng/ml]^4)^	10.26 (9.22–11.31)		11.62 (9.99–13.25)		ns^5)^
hs-CRP, [mg/l]^4)^	2.76 (2.08–3.44)		3.14 (2.43–3.85)		ns^5)^
TNF-α, [pg/ml]^4)^	11.03 (4.28–17.78)		11.57 (3.69–19.46)		ns^5)^
IL6, [pg/ml]^4)^	3.53 (2.05–5.01)		4.35 (2.15–6.56)		ns^5)^

^1)^The long-term life dissatisfaction burden score is the sum of life satisfaction scores in 1998, 1999, 2001 and 2005.

^2)^Missing 6 cases; ^3)^Fisher’s exact test; ^4)^missing 23 cases; ^5)^Mann–Whitney U-test.

*Abbreviations*: *NSAID* Non-steroidal anti-inflammatory drug, *BMI* Body mass index, *hs-CRP* High-sensitivity C-reactive protein, *TNF-α* Tumor Necrosis Factor alpha, *IL6* Interleukin 6.

The anti-inflammatory cytokine adiponectin was significantly lower among the long-term dissatisfied (p = 0.014) compared to the long-term satisfied. The levels of proinflammatory markers TNF-α, IL-6, resistin and hs-CRP were also higher in the long-term satisfied, but statistical significance was not reached. In further analyses, no significant association was found between the use of statins (yes vs. no) and the inflammatory markers, i.e. the level (mean; 95% confidence intervals) of adiponectin [μg/ml] (14.3; 10.5–18.1 vs. 16.9; 14.9–19.0; Z = −1.76; p = ns), resistin [ng/ml] (10.4; 8.6–12.3 vs. 11.1; 10.0–12.2; Z = −0.29; p = ns), hs-CRP [mg/l] (3.1; 1.9–4.3 vs. 2.9; 2.4–3.4; Z = −0.64; p = ns), IL-6 [pg/ml] (4.3; 1.2–7.5 vs. 3.8; 2.4–5.2; Z = −0.09; p = ns) and TNF-α [pg/ml] (7.3; 1.2–13.4 vs. 12.4; 6.0–18.7; Z = −0.12; p = ns). The same pattern was seen when those categorized as stable dissatisfied were compared with those with stable satisfaction (data not shown).

According to the additional analyses, no significant association was found between the cancer diagnosis (yes vs. no) and the inflammatory markers, i.e. the level (mean; 95% confidence intervals) of adiponectin [μg/ml] (15.2; 8.8–39.2 vs. 15.9; 14.2–17.7; Z = −1.03; p = ns), resistin [ng/ml] (14.9; 0.8–29.4 vs. 10.8; 9.9–11.8; Z = −0.36; p = ns), hs-CRP [mg/l] (2.5; 0.3–5.4 vs. 3.0; 2.5–3.5; Z = −0.36; p = ns), IL-6 [pg/ml] (2.5; 1.8–6.9 vs. 4.0; 2.6–5.3; Z = −0.39; p = ns) and TNF-α [pg/ml] (2.5; 0.5–5.5 vs. 11.5; 6.2–16.7; Z = −0.21; p = ns).

According to both bivariate and multivariate linear regression analysis, poor sleep, living alone, current smoking, and especially poor current social support were significantly associated with long-term life dissatisfaction (logLSburden) (Table 1). The same applied for metabolic syndrome, the use of statins and low serum levels of adiponectin (Table 2). On the other hand, age, gender, alcohol consumption, coronary heart disease, rheumatoid arthritis, the use of oral corticosteroids or NSAIDs, body mass index, and the serum proinflammatory markers resistin, hs-CRP, TNF-α, and IL-6 were not significantly related to logLSburden.

When correlation between continuous variables were tested, age (Pearson 0.141, p = 0.014; Spearman 0.156, p = 0.006) and adiponectin (Pearson 0.139, p = 0.016; Spearman 0.133, p = 0.021) were significantly correlated with logLSburd, while body mass index (Pearson 0.099, p = ns; Spearman 0.081, p = ns) and proinflammatory markers hS-CRP (Pearson 0.043, p = ns; Spearman 0.100, p = ns); resistin (Pearson 0.021, p = ns; Spearman 0.059, p = ns); IL6 (Pearson 0.038, p = ns; Spearman 0.072, p = ns) and TNF-α (Pearson 0.043, p = ns; Spearman 0.072, p = ns) were not.

In the final linear regression model (Table 3), including all the above-mentioned significant variables from multivariate models 1 and 2, poor social support (B =0.138; Beta = 0.315; t = 6.19; p < 0.001), marital status (i.e. living alone) (B = 0.049; Beta = 0.117; t = 2.350; p = 0.019), smoking (B = 0.087; Beta = 0.227; t = 4.575; p < 0.001), poor sleep (B = 0.052; Beta = 0.174; t = 3.465; p = 0.001, use of statins (B = −0.052; Beta = −0.152; t = −3.076; p = 0.002) and adiponectin (B = −0.001; Beta = −0.104; t = −2.070; p = 0.039) were independently associated with higher life dissatisfaction burden score (logLSburden) whereas association of metabolic syndrome was marginally insignificant (B = 0.029; p = 0.055).

## Discussion

The present study investigated the significant independent correlates of the long-term dissatisfaction burden from a set of sociodemographic and health-related factors, also including several biomarkers. To the best of our knowledge, this is the first study on a general population sample to present lowered serum adiponectin levels in individuals experiencing long-term life dissatisfaction. This was true also after using life dissatisfaction burden as a continuous score in a multivariate linear regression model including significant sociodemographic and health-related factors such as BMI, metabolic syndrome and the use of statins. Still, poor social support was the strongest correlate of the long-term life dissatisfaction burden, which is consistent with previous research [11,46]. This was true independent from marital status, a significant correlate, too. Poor sleep was also an independent correlate of life dissatisfaction in the model including adiponectin, but it has also been reported to have its own predictive ability in relation to inflammation processes [16,17,20].

Chronic psychological stress, in general, has been linked with inflammation [18], for instance through elevated levels of IL-6 and hs-CRP [19,20]. This was not verified in our data with statistical significance, but our findings suggest that the long-term life dissatisfaction burden presents a form of psychological stress, which may modulate inflammation processes by reducing the levels of the anti-inflammatory marker adiponectin. However, more research on this is needed with larger general population samples and with longitudinal and more detailed data, which would allow further study of the mechanisms behind these associations.

The health status is strongly associated with life satisfaction [47]. The long-term life dissatisfaction burden may compromise somatic health in various ways. Current smoking in addition to poor sleep and not living in a partnership were associated with long-term life dissatisfaction. On the other hand, inflammatory pathogenesis involved in metabolic syndrome can also affect the central nervous system, even predisposing individuals to brain dysfunction [48]. Thus, its role should also be investigated further in relation to global well-being. In the present study, metabolic syndrome was marginally not a significant factor in the final model, and it did not explain the observed relationship between adiponectin and long-term life dissatisfaction.

The use of statins was significantly less frequent among individuals with long-term life dissatisfaction, even though they had a higher prevalence of metabolic syndrome. This might suggest poor somatic health care among the dissatisfied. Previously, a decreasing level of health care has been reported in relation to an increasing severity of mental disorders [49], but causality in our findings cannot be verified. The psychosocial factors linked with life dissatisfaction resemble those previously found with cardiovascular disease [50,51]. Indeed, life satisfaction has been significantly linked with a decreased risk of coronary heart disease [6], which is in line with the present study.

Health behavior also matters. Inflammation has been observed to be lower in physically active adults [52], and moderate physical training can modulate the cytokine profile and improve the quality of sleep [53]. On the other hand, smoking seems to increase low-grade inflammation, e.g. IL-6 levels [54]. We have previously observed that smoking is independently associated with long-term life dissatisfaction [42]. Thus, all these associations between long-term life dissatisfaction, health behavior, and metabolic syndrome, as well as cardiovascular risk factors, should increase the knowledge available to the physician in preventing adverse somatic consequences among those with low subjective well-being.

### Strengths and limitations

A strength of this study was its community sample and the availability of several important health-related measures, including biological markers, for assessing the factors related to the long-term life dissatisfaction burden [55]. The multivariate analyses as well as taking to account of the role of the body mass index and use of statins helped to control for potential bias. The longitudinal assessment of life satisfaction provided valuable information on cumulative burden of dissatisfaction, while also measuring aspects of positive mental health. Due to the somewhat small sample size and considerable stability of life satisfaction in the long-term perspective, the changes in life satisfaction over time were investigated to a limited extent with respect to the identified correlates. A more detailed disease history and longitudinal data on health-related factors of the participants would have been a benefit and should be covered in the future research.

## Conclusions

Social and health behavioural factors along with stress and inflammation play a role in life dissatisfaction, but further research is needed to verify the biological associations found in this study. Several correlates of long-term life dissatisfaction resembled common risk factors for somatic diseases, supporting the close relationships between subjective well-being and somatic morbidity.

## Competing interests

The authors declare that they have no competing interests.

## Authors’ contributions

TR, SML, HK-H and HV designed the study and wrote the manuscript. TR and HK-H managed and conducted the statistical analyses and interpreted the data by the aid of biostatistician KH, TS, HK-H, JH and HV collected the data. All authors contributed to and have approved the final manuscript.

## Pre-publication history

The pre-publication history for this paper can be accessed here:

http://www.biomedcentral.com/1471-244X/13/202/prepub
